# Pulmonary carcinosarcoma initially presenting as invasive aspergillosis: a case report of previously unreported combination

**DOI:** 10.1186/1746-1596-5-11

**Published:** 2010-01-29

**Authors:** Ariyo O Olobatoke, Doina David, Wasif Hafeez, Thien Van, Husain A Saleh

**Affiliations:** 1 Department of Medicine, Sinai Grace Hospital/Detroit Medical Center, Detroit, Michigan, USA; 2 Department of Pathology, Wayne State University and Detroit Medical Center/Sinai Grace Hospital Detroit Michigan, USA; 3 Department of Medicine, Division of Infectious Diseases, Sinai Grace Hospital/Detroit Medical Center, Detroit, Michigan, USA

## Abstract

Carcinosarcoma of the lung is a malignant tumor composed of a mixture of carcinoma and sarcoma elements. The carcinomatous component is most commonly squamous followed by adenocarcinoma. The sarcomatous component commonly comprises the bulk of the tumor and shows poorly differentiated spindle cell features. Foci of differentiated sarcomatous elements such as chondrosarcoma and osteosarcoma may be seen. Aspergillus pneumonia is the most common form of invasive aspergillosis and occurs mainly in patients with malignancy, immunocompromizing or debilitating diseases. Patients with Aspergillus pneumonia present with fever, cough, chest pain and occasionally hemoptysis. Tissue examination is the most reliable method for diagnosis, and mortality rate is high.

We describe a case of primary carcinosarcoma of the lung concurrently occurring with invasive pulmonary aspergillosis in a 66-year old patient.

## Background

Primary carcinosarcoma of the lung is exceedingly rare [1-8]. In the new World Health Organization (WHO) classification of lung tumors, it is described as malignancy composed of a mixture of carcinoma and sarcoma elements. The sarcomatous is usually spindle cell but may contain cartilage, bone or skeletal muscle components. However, controversy exists in the classification of this tumor and some authors may include sarcomatoid carcinoma in this category.

Invasive pulmonary aspergillosis is a spectrum of reactions that depend on a combination of patient immunologic status, underlying lung condition and the nature of exposure to aspergillus fungus. It most often presents as aspergillus pneumonia and almost always involves immunoecompromized or debilitated patients with underlying malignancy [9]. Acute leukemia patients are very susceptible particularly during times of neutropenia. Patients with cirrhosis, chronic obstructive pulmonary disease (COPD), autoimmune deficiency syndrome (AIDS) and prolonged steroid treatment are at increased risk. Here we report a case of primary pulmonary carcinosarcoma with synchronous aspergillous pneumonia in a patient with previous prostate cancer. On review of the literature, this combination has not been reported before.

## Case Report

A 66 years old African American man presented to the hospital with 1 week history of progressive shortness of breath and bilateral calf pain. He complained of occasional productive cough but denied any chest pain, hemoptysis, night sweats, palpitation, or dyspnea. He had a history of peripheral vascular disease and prostate cancer Gleason's score 6(3+3) about 8 years ago for which he had prostatectomy and subsequent penile implant for erectile dysfunction. He had an extensive smoking history but no alcohol or street drug abuse. Furthermore, he had a prior 8-year history of incarceration and a family history of lung cancer.

**Figure 1:**
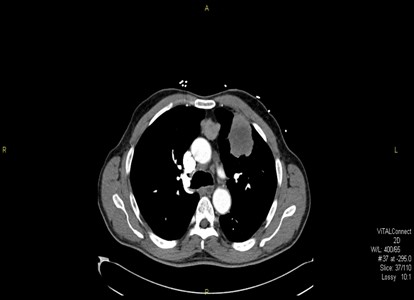
**A CT scan with contrast of the chest showing large left upper lobe lung mass involving the pleural surface**.

Due to his chest symptoms, he had a chest x-ray followed by Computerized Tomography (CT) scan of the chest which showed a left upper lung mass (4.5 × 5.5 × 5 cm) with mediastinal and right hilar adenopathy [Fig.1]. No pleural or pericardial effusion was noted. CT of the head and bone scan revealed no metastasis.

**Figure 2:**
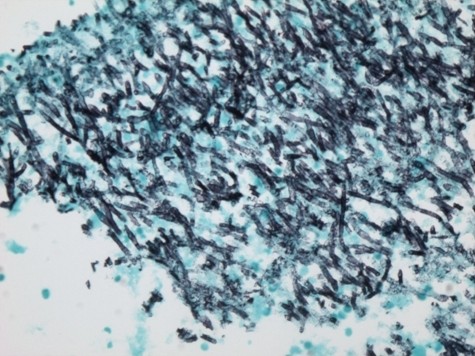
**A GMS stain showing aspergillus fungal hyphae with uniform septated hyphae, and branching at 45 degrees (×100, Gomori Methanamine Silver stain)**.

A CT guided fine needle aspiration cytology of the left lung mass showed inflammatory necrotic background with several large aggregates of fungi. On Gomori Methanamine Silver (GMS) stain, the hyphae had uniform diameter, septation and branching at 45 degree, morphologically compatible with aspergillus species [Fig. 2]. A special stain for Acid Fast Bacilli (AFB) was negative, and no tumor cells were identified. Based on these findings, he was commenced on liposomal Amphotericin B for 2 weeks followed by Voriconazole to complete a 6 week course of antifungal therapy for pulmonary aspergillosis. His hemoglobin was 7.7 g/dl, white blood cell count 7.7 k/mm^3^, and absolute neutrophil, monocyte and lymphocyte count of 4.6 k/mm^3^, 0.6 k/mm^3 ^and 3.2 k/mm^3 ^respectively. Serum creatinine was 1.4 mg/dl and blood urea nitrogen 14 mg/dl. HIV and Hepatitis C serology were negative. He improved and was discharged on voriconazole. However, he presented again after about 8 weeks with new onset hemoptysis and night sweats. He subsequently had bronchoscopy with bronchoalveolar lavage (BAL) which returned negative for mycobacterium, fungus, legionella and cytomegalovirus on culture. Direct Fluorescent Antibody of BAL fluid was negative for Parainfluenza 1, Adenovirus, Herpes Simplex I&II, Respiratory Syncytial Virus, Varicella Zoster Influenza A&B and Adenovirus. BAL fluid was negative for malignant cells and Pneumocystis carinii.

Pulmonary function test showed an obstructive pattern (FEV1/FVC ratio 58% of reference). He subsequently had a thoracotomy with a left upper lobectomy revealing biphasic malignant tumor (carcinosarcoma).

### Pathology description

**Figure 3:**
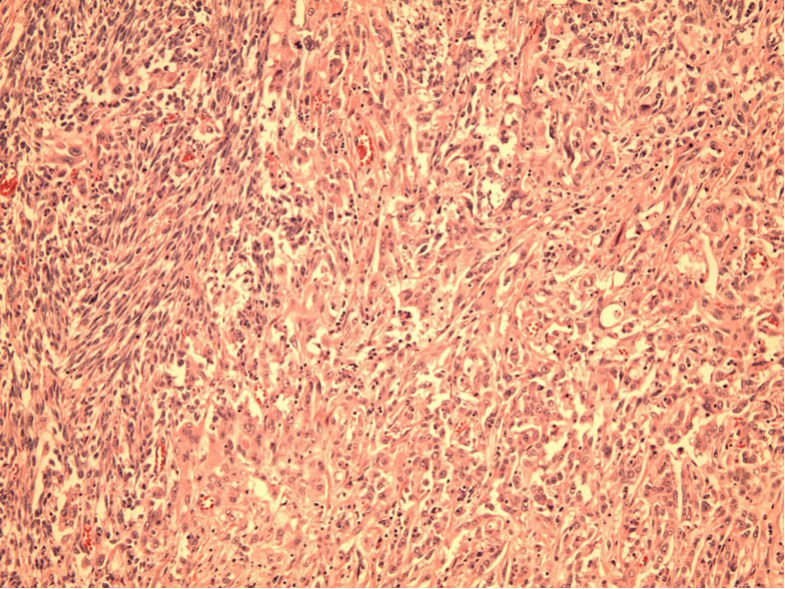
**Carcinosarcoma with areas of sarcomatous spindle cell proliferation (left) and an epithelial carcinoma component (right)**. (×100, Hematoxylin and Eosin).

**Figure 4:**
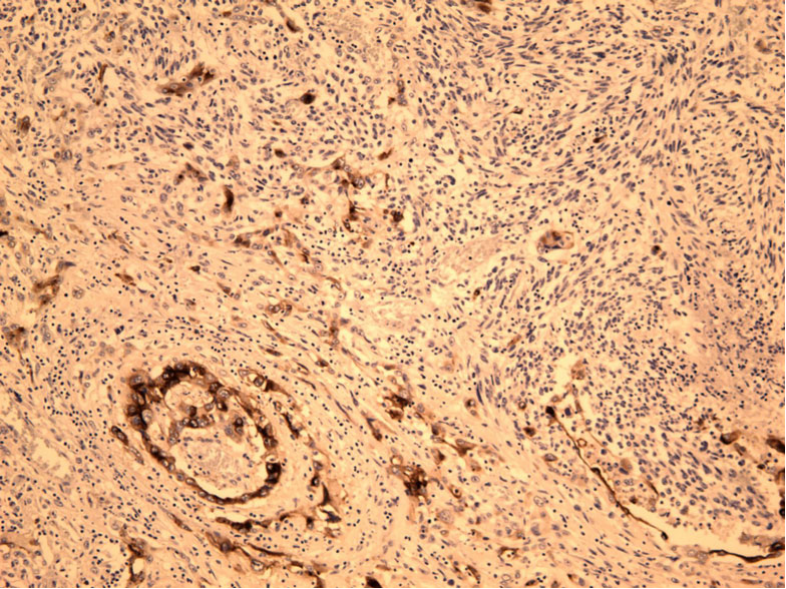
**Epithelial carcinoma component of the tumor positive for cytokeratin AE1/AE3 immunostain (×100)**.

**Figure 5:**
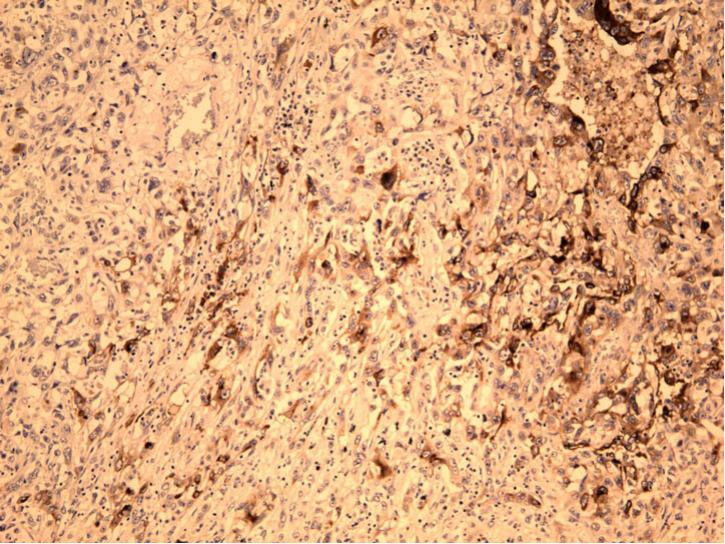
**Epithelial carcinoma component of the tumor showing positivity for epithelial membrane antigen immunostain (EMA) (×100)**.

**Figure 6:**
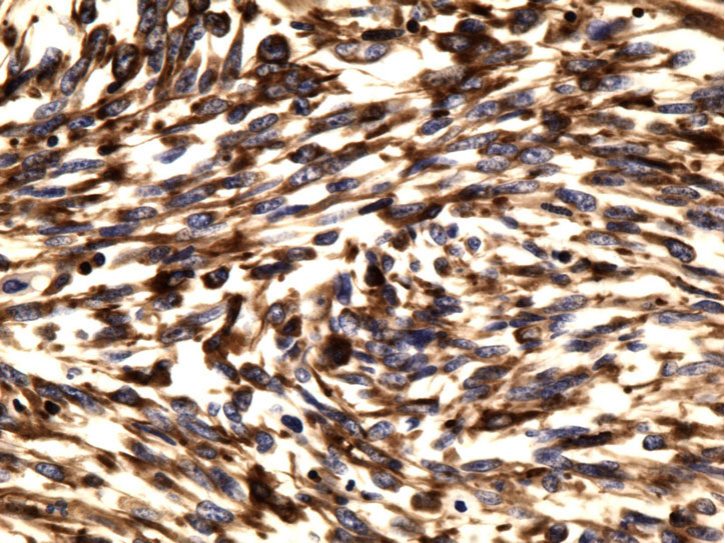
**Sarcoma component of the tumor showing spindle tumor cells positive for vimentin (×400)**.

A left upper lobectomy (20 × 15.5 × 5.5 cm) was done. Sectioning revealed a large tan-white circumscribed partly hemorrhagic mass with central necrotic cavity. The mass was abutting the pleural surface and measured 8.5 × 6.5 × 5.5 cm of which intra-operative frozen section was diagnosed as poorly differentiated squamous cell carcinoma. Interestingly, final surgical pathology examination revealed a poorly differentiated biphasic malignant neoplasm with epithelial and spindle cell components and necrosis [Fig. 3]. The carcinomatous component showed predominantly squamous cell differentiation with foci of aborted glandular structures. The sarcomatous component displayed interlacing short fascicles of malignant spindle cells with areas of marked cellular pleomorphism and bizarre giant tumor cells. Numerous atypical mitoses and large areas of geographic necrosis were evident. Morphologically, the differential included poorly differentiated lung carcinoma with "sarcomatoid" growth pattern, primary pulmonary carcinosarcoma and biphasic poorly differentiated synovial sarcoma. The tumor involved the pleural surface and the surgical bronchial margin. An extensive panel of immunostains was performed for further classification and more precise diagnosis. The epithelial carcinomatous tumor cells showed strong immunoreactivity with pankeratin AE1/AE3 (Fig. 4) and epithelial membrane antigen (EMA) (Fig. 5) and focally for P63; while vimentin stain was strongly positive in the spindle cell component but not in the carcinomatous component [Fig. 6]. Patchy foci of spindle tumor cells were reactive for calponin and smooth muscle actin. All other markers including desmin, cKit (CD117), S100 protein, CD 34, HMB45, bcl2, and CD99 were negative essentially excluding synovial sarcoma. Also, Fluorescence In-situ Hybridization (FISH) analysis did not detect t(X;18) translocations of the SYT/18q11.2 region that are characteristic of synovial sarcoma.

**Figure 7:**
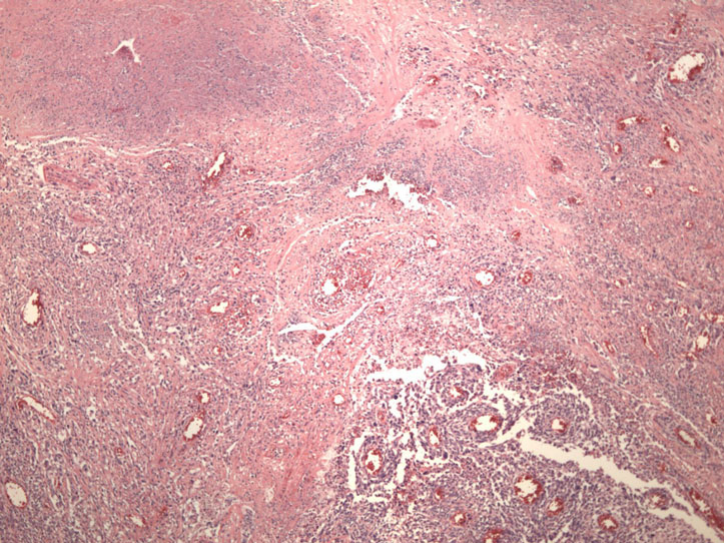
**An area of the tumor showing hemorrhagic infarction and shadow outlines of destructed blood vessels (×100, Hematoxylin and Eosin)**.

**Figure 8:**
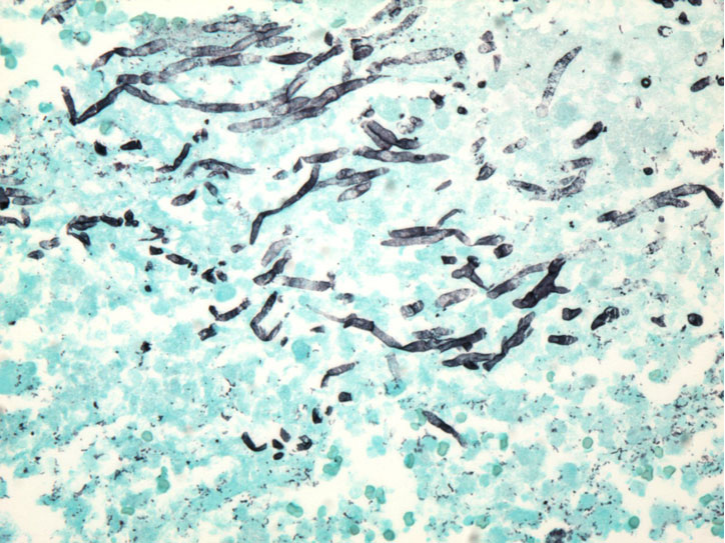
**Groups of aspergillus fungal hyphae are present in an area of hemorrhagic infarction in the resected specimen (×400, Gomori Methanamine Silver stain)**.

Within the resection specimen areas of infarction and coagulative necrosis were identified, some of them bordering viable tumor (Fig. 7). In one such area, fungal hyphae morphologically consistent with aspergillus species were seen and confirmed by GMS fungal stain (Fig. 8). Furthermore, shadow outlines of destructed blood vessels were noted in the infarcted areas.

Additionally, intraoperatively obtained pleural fluid showed many malignant cells arranged in sheets and single cell pattern. The tumor cells were round to spindle and highly pleomorphic. Immunostains for CK 5/6, CK 7, CK 20, TTF-1, CD 15 and CD 30 were all negative in the tumor cells.

Unfortunately, our patient had a complicated postoperative course in the intensive care unit. He developed pneumonia with Acinetobacter and was difficult to wean him from the ventilator. He finally succumbed to cardiac arrest.

## Discussion

Pulmonary carcinosarcoma is a malignant tumor with a mixture of carcinoma and sarcoma [1,2]. In the WHO classification of lung tumors, it is included in a group of poorly differentiated non-small cell lung carcinomas that contain a component of sarcomatoid differentiation, so called sarcomatoid carcinoma. The average age of diagnosis is 60 years with men to women ratio of 4:1 and more than 90% of these patients have a history of heavy smoking [2]. Pulmonary carcinosarcoma accounts for < 1% of all primary pulmonary neoplasms [6].

The carcinomatous component is more often squamous cell carcinoma, followed by adenocarcinoma and large cell carcinoma; whereas the most common mesenchymal component is poorly differentiated spindle cell sarcoma. Nevertheless, foci of rhabdomyosarcoma, osteosarcoma, and chondrosarcoma are often found [3].

In a retrospective analysis of 2,400 lung cancer patients between 1975 and 1995 conducted by Huwer H et al, only seven patients (0.3%) had pulmonary carcinosarcoma [4]. Diaconita reported eight cases among 3000 patients with malignant pulmonary tumors [5].

Immunohistochemical (IHC) staining enhances the distinction of carcinomatous from sarcomatous components within the tumor. When heterologous sarcoma elements such as cartilage or skeletal muscle are present, it is easier to confirm the biphasic nature of the tumor, although immunostains can be of further help such as Myogenin and Myo D1 (rhabdomyosarcoma); smooth muscle actin and desmin (leiomyosarcoma); and S100 (chondrosarcoma). However, carcinosarcomas are difficult to diagnose preoperatively. Biopsy of the tumor, especially when centrally located, often shows only one component, and peripheral tumors are difficult to reach endoscopically [4].

Histogenetically, Carcinosarcomas may represent malignant epithelial neoplasms undergoing divergent tissue differentiation originating from a single clone [7]. The prognosis of these patients is unfavorable based on a small number of cases described in literature. Ishida et al reported a 9-month median survival of 5 patients [8]. Davis et al reported a 12 month median survival time in 15 patients post resection [6]. Huwer et al reviewed seven patients with pulmonary carcinosarcoma out of 2,400 cases of lung cancers and found that mortality was related to tumor recurrence or distant metastasis of the sarcoma component [4]. In a clinicopathologic study, Koss et al retrospectively reviewed 66 patients with pulmonary carcinosarcoma from the archives of the Armed Forces Institute of Pathology (AFIP), and compared them to 33 cases of pleomorphic carcinoma and found that carcinosarcomas had poor prognosis with a 5-year survival rate of 21.3%. Of several clinical and pathologic parameters, only tumor size (6 cm or more) appeared to be related to reduced survival (p = 0.0195) [3].

Invasive Aspergillosis, is a major cause of morbidity and mortality in the severely immunocompromised. Risk factors include prolonged and severe neutropenia, hematopoietic stem cell and solid organ transplantation, advanced AIDS, and chronic granulomatous disease [9]. It is rarely found in patients with solid tumors and in the absence of the afore mentioned major risks factors. In retrospective microbiology studies of patients with "proven" and "probable" invasive aspergillosis based on the European Organization for Research and Treatment of Cancer, and the Mycosis Study Group of the National Institute of Allergy and Infectious Diseases criteria between 1993-2003, only 1 of a total of 13 patients who met the criteria for invasive aspergillosis was found to have lung cancer [10-12]. Steroid use and lymphopenia were common risk factors among the patients with solid tumors and invasive aspergilosis [10]. An autopsy review at M. D. Anderson Caner Center of 588 patients with hematologic malignancies and 144 patients with solid tumors done between 1993-2002 revealed that 102 (17.3%) had hematologic malignancies, while only 1 patient (0.68%) had solid tumor (p < 0.01) [10]. The findings of this studies, as in our case, are in agreement with previous studies that invasive aspergillosis is much more common in patients with hematologic than in solid malignancies.

Nivoix Y et al retrospectively analyzed 289 patients with presentation that fulfilled the criteria for possible, probable, or proven invasive aspergillosis according to the international definitions. The predictors of increased overall mortality were in patients who received allogenic hematopoietic stem cell or solid-organ transplant, prior respiratory disease, corticosteroid therapy, renal impairment, low monocyte counts, disseminated aspergillosis, diffuse pulmonary lesions, pleural effusion, and proven or probable aspergillosis [11].

In our case, the invasive aspergillosis and the subsequently diagnosed carcinosarcoma occupied the same location of the left upper lobe. The typical tissue reaction in aspergillus pneumonia is known to be hemorrhagic infarction with a sparse inflammatory infiltrate [13], and our case revealed similar features. In addition, despite complete course of antifungal therapy, occasional foci of residual aspergillus hyphae were identified in such areas of hemorrhagic infarction.

In conclusion, the combination of invasive aspergillosis and solid malignancies is an extremely rare occurrence. To the best of our knowledge, there has not been any reported case of pulmonary carcinosarcoma and invasive aspergillosis in the literature. Furthermore, this case report is significant because the patient did not have any of the commonly described risk factors for developing invasive aspergillosis. Limited studies found in literature have shown poor prognosis in patients with either invasive aspergillosis or pulmonary carcinosarcoma. Whether the coexistence of these two entities in a patient increases mortality remains to be determined in case series, retrospective or prospective studies.

## Consent

Written informed consent was obtained from the patient for publication of this case report and accompanying images. A copy of the written consent is available for review by the Editor-in-Chief of this journal.

## Competing interests

The authors declare that they have no competing interests.

## Authors' contributions

AO provided the clinical data and drafted the manuscript, DD described the pathology component and took photographs, participated in writing the discussion, coordinated and edited the manuscript, WH edited the clinical part of the manuscript, TV provided part of the references and edited the clinical case presentation, HS reviewed the entire manuscript, participated in writing the discussion and the pathology component and edited the manuscript. All authors read and approved the final manuscript.
